# Clinical Efficacy of Chemotherapy Regimen Combined with Levofloxacin in Patients with Pulmonary Tuberculosis Complicated with Type-2 Diabetes

**DOI:** 10.12669/pjms.39.2.6364

**Published:** 2023

**Authors:** Pei-pei Luo, Li Wu, Fang Liu, Yan-hong Tian, Lai-yin Chen, Ya-lin Liu

**Affiliations:** 1 Pei-pei Luo, Department of Respiratory, Baoding people’s Hospital, Baoding 071000, Hebei, China; 2 Li Wu,Department of Infection, Baoding people’s Hospital, Baoding 071000, Hebei, China; 3 Fang Liu, Department of Infection, Baoding people’s Hospital, Baoding 071000, Hebei, China; 4 Yan-hong Tian, Department of Infection, Baoding people’s Hospital, Baoding 071000, Hebei, China; 5 Lai-yin Chen, Department of Infection, Baoding people’s Hospital, Baoding 071000, Hebei, China; 6 Ya-lin Liu, Department of Infection, Baoding people’s Hospital, Baoding 071000, Hebei, China

**Keywords:** Combined chemotherapy, Levofloxacin, Pulmonary tuberculosis, Type-2 diabetes, Treatment

## Abstract

**Objective:**

To evaluate the clinical efficacy of a chemotherapy regimen combined with levofloxacin in patients with pulmonary tuberculosis complicated with Type-2 diabetes.

**Methods:**

Total 80 patients with pulmonary tuberculosis complicated with Type-2 diabetes admitted to Baoding People’s Hospital from January, 2019 to January, 2022 were randomly divided into two groups: the experimental group and the control group, with 40 cases in each group. Patients in the control group were given the conventional 2HRZE/10HRE regimen, while those in the experimental group were given the chemotherapy regimen 2HRZEL/6HRE combined with levofloxacin. Sixty four slice spiral CT was used for chest plain scan before and after treatment, respectively, to evaluate the absorption of lesions based on the range of lung lesions; Venous blood was drawn to detect the changes of oxidative stress indicators, the incidence of adverse drug reactions and the negative conversion rate of sputum tuberculosis bacteria in the two groups.

**Results:**

After treatment, the efficacy of the experimental group was 90%, which was significantly higher than that of the control group (67.5%), with a statistically significant difference (p=0.01). After treatment, CD3^+^, CD4^+^, CD4^+^/CD8^+^ and other indicators in the experimental group were significantly higher than those in the control group, with a statistically significant difference (CD3^+^, p=0.01; CD4^+^, p=0.01; CD4^+^/CD8^+^, p=0.00), while CD8+ did not change significantly (p=0.92); The incidence of adverse reactions was 52.5% in the experimental group and 47.5% in the control group, with no statistically significant difference (p=0.66); The negative conversion rate of patients in the experimental group was significantly higher than that in the control group at one month, three months and six months after treatment, with a statistically significant difference (p<0.05).

**Conclusion:**

Chemotherapy combined with levofloxacin is a safe and effective regimen for patients’ pulmonary tuberculosis complicated with Type-2 diabetes, boasting a variety of benefits such as improved clinical efficacy, ameliorated cellular immune status, a high negative conversion rate of sputum tuberculosis bacteria, and no significant increase in adverse reactions.

## INTRODUCTION

Tuberculosis (TB) has been coexisting with the development of human history since the Stone Age. It is still one of the chronic infectious diseases that threaten human health and is the top 10 leading causes of human death worldwide.^1^ TB is more common in developing countries and poverty-stricken areas, especially in immunocompromised patients.^2^ Studies have shown that^3^ the incidence of pulmonary TB combined with diabetes is increasing year by year, which has become a global health problem. It was suggested in the study of Deshmukh et al.^4^ that compared with the normal population, patients with pulmonary TB have a higher probability of suffering from diabetes, while patients with pulmonary TB complicated with diabetes have significantly increased blood and tissue glucose levels due to abnormal glucose metabolism caused by diabetes so that the normal immune function of cells is inhibited, thus accelerating the proliferation of mycobacterium TB and aggravating pulmonary TB. It has been reported in the literature^5^ that patients with pulmonary TB complicated with diabetes have rapid disease progression, a high positive rate of sputum culture, more cavities, difficult treatment and poor clinical efficacy. To this end, correct anti-TB drugs should be selected and appropriate chemotherapy regimens should be developed to treat pulmonary TB complicated with diabetes. Studies have shown that^6^ levofloxacin is a broad-spectrum antibacterial drug with a strong antibacterial effect. Levofloxacin can bind to bacterial DNA gyrase subunit A for rapid bactericidal action by inhibiting bacterial DNA replication and DNA helix activity. In this study, a short-course chemotherapy regimen combined with levofloxacin was used for patients with pulmonary TB complicated with Type-2 diabetes, and certain clinical effects were achieved.

## METHODS

Eighty patients with pulmonary TB complicated with Type-2 diabetes admitted to Baoding People’s Hospital from January 2019 to January 2022 were randomly divided into two groups: the experimental group and the control group, with 40 cases in each group. Among them, there were 27 males and 13 females in the experimental group, aged 32-68 years, with an average of 52.70±12.18 years, and 25 males and 15 females in the control group, aged 27-70 years, with an average of 51.86±12.84 years. No significant difference was observed in the comparison of general data between the two groups, which was comparable (Table-I). The study was approved by the Institutional Ethics Committee of Baoding people’s Hospital on September 17, 2020(No.:2020020), and written informed consent was obtained from all participants.

**Table-I:** Comparative analysis of general data between the experimental group and the control group (*χ̅*±*S*) n=40.

Indicators	Experimental group	Control group	t/χ^2^	p
Age (years old)	52.70±12.18	51.86±12.84	0.30	0.76
Male (cases %)	27 (67.5%)	25 (62.5%)	0.22	0.64
Course of diabetes (years)	7.12±2.33	7.31±2.74	0.33	0.74
***Accompanied symptoms***				
Cough	24 (60%)	22 (55%)	0.20	0.65
Chest pain	6 (15%)	7 (17.5%)	0.09	0.76
Fever	23 (57.5%)	25 (62.5%)	0.21	0.65
Other	9 (22.5%)	7 (17.5%)	0.31	0.58
Smoking history (%)	24 (60%)	27 (67.5%)	0.49	0.49

p>0.05.

### Inclusion criteria:

- Patients who met the diagnostic criteria for TB^7^ and Type-2 diabetes^8^ and whose chest imaging examinations (CT, X-ray) could accurately calculate the size of lung lesions;
- Patients under the age of 70;
- Patients who have not received regular anti-TB treatment in the past;
- Patients without serious cardiovascular and cerebrovascular diseases, liver and kidney disease history, and without extrapulmonary TB;
- Patients without HIV/AIDS;
- Patients without obvious disturbance of consciousness and able to cooperate with the completion of the study;
- Patients who have not used drugs that affect the study recently, such as immunosuppressants and hormone drugs;
- Patients who are not allergic to the drugs involved in the study;
- Patients who signed the consent form by themselves and their family members and were able to cooperate with the study.

### Exclusion Criteria:

- Children and pregnant women;
- Patients with drug-resistant TB;
- Patients who cannot tolerate the drugs used in the study protocol for various reasons;
- Patients with long QT interval (>480 ms);
- Patients with mental or consciousness disorders, cognitive disorders, and unable to cooperate with the study;
- Patients with allergies, intolerance or contraindications to the relevant drugs involved in the study.

Patients in the control group were given the conventional 2HRZE/10HRE regimen: oral isoniazid tablets 0.3g, qd; rifampicin 0.45g, qd; pyrazinamide 1.5g, qd; ethambutol hydrochloride Tablet 0.75g, qd. After two months of treatment, patients were switched to the HRE regimen for ten months,^9^ with the drug application method and dose remaining unchanged. Patients in the experimental group were given the chemotherapy regimen combined with levofloxacin: 2HRZEL/6HRE, isoniazid 0.3g daily at a draught; rifampicin 0.45g daily at a draught; pyrazinamide 0.5g daily, tid; ethambutol 0.75 g daily at a draught; levofloxacin 0.8g daily, orally.^10^ All patients underwent sputum culture + drug sensitivity test before treatment. After two months of treatment, patients were switched to the HRE regimen for six months, with the drug application method and dose as above.

### Observation indicators:

### Evaluation of clinical efficacy:

Siemens 64-slice spiral CT was used for chest plain scan before and 3 months after treatment to evaluate the absorption of lesions based on the range of lung lesions.^11^ Remarkably effective: ≥50% reduction in the range of lesions compared with before treatment; Effective: 20%-50% reduction in the range of lesions compared with before treatment; Invalid: ≤20% reduction in the range of lesions compared with before treatment; Deterioration: Enlarged or disseminated range of lesions. Total effective rate = (remarkably effective + effective)/total number of cases;

### Analysis of immune status:

Fasting blood was taken in the morning before and after treatment, respectively, to detect the levels of T lymphocyte subsets CD3+, CD4+, CD8+, CD4+/CD8+, and the differences between the two groups before and after treatment were compared

### Assessment of adverse drug reactions:

Adverse drug reactions of the two groups after treatment were recorded, including: abnormal liver function, neuritis, abnormal renal function, rash, leukopenia, gastrointestinal reactions, etc.

### Comparative analysis of the negative

*conversion rate of sputum mycobacterium TB:* After treatment, patients were examined for sputum bacteria every month. If the examination for more than two consecutive months shows that sputum bacteria turn negative and there is no recurrence, it is regarded as successful negative conversion.

### Statistical Analysis:

All data in this study were analyzed with SPSS 20.0 software, and measurement data were expressed as (±S). Data between the experimental group and the control group were analyzed using two independent *t* test. Paired *t* test was utilized for the comparative analysis of each indicator in the experimental group before and after treatment. P<0.05 indicates a statistically significant difference.

## RESULTS

The efficacy of the experimental group was 90%, which was significantly higher than that of the control group (67.5%), with a statistically significant difference (p=0.01, see Table-II). No significant difference was observed in the levels of CD3^+^, CD4^+^, CD8^+^ and CD4^+^/CD8^+^ before treatment of the two groups (p>0.05). After treatment, CD3^+^, CD4^+^, CD4^+^/CD8^+^ and other indicators in the experimental group were significantly higher than those in the control group, with a statistically significant difference (CD3^+^, p=0.01; CD4^+^, p=0.01; CD4^+^/CD8^+^, p=0.00), while CD8^+^ did not change significantly (p=0.92) (Table-III).

**Table-II:** Comparative analysis of clinical efficacy between the two groups (*χ̅*±*S*) n=40.

Group	Remarkably effective	Effective	Invalid	Deterioration	Effective rate
Experimental group	27	9	3	1	36 (90%)
Control group	22	5	9	4	27 (67.5%)
c^2^					6.05
p					0.01

p<0.05

**Table-III:** Comparative analysis of the levels of T lymphocyte subsets between the two groups before and after treatment (*χ̅*±*S*) n=30.

Indicators	Observation points	Experimental group	Control group	t	p
CD3+ (%)	Before treatment	42.02±6.73	42.13±6.55	0.07	0.94
	After treatment*	48.82±5.84	45.27±5.47	2.81	0.01
CD4+ (%)	Before treatment	25.82±5.21	25.36±4.74	0.41	0.68
	After treatment*	36.18±6.39	32.74±5.77	2.53	0.01
CD8+ (%)	Before treatment	23.07±3.57	23.04±3.86	0.05	0.97
	After treatment	24.16±3.85	24.08±3.35	0.09	0.92
CD4+/CD8+	Before treatment	1.47±0.62	1.51±0.43	0.34	0.73
	After treatment*	1.85±0.31	1.50±0.46	3.99	0.00

* p<0.05

Comparative analysis of the incidence of adverse drug reactions between the two groups after treatment showed that the incidence of adverse reactions was 52.5% in the experimental group, which was higher than that of the control group (47.5%), with no statistically significant difference (p=0.66) (Table-IV).

**Table-IV:** Comparative analysis of adverse drug reactions between the two groups after treatment (*χ̅*±*S*) n=40.

Group	Rash	Gastrointestinal reaction	WBC decrease	Renal impairment	Neuritis	Liver damage	Incidence
Experimental group	2	3	4	3	3	6	21 (52.5%)
Control group	4	3	5	0	3	4	19 (47.5%)
c^2^							0.20
p							0.66

p>0.05

The comparative analysis of the negative conversion rate of sputum tuberculosis bacteria between the experimental group and the control group after treatment suggested that the negative conversion rate of patients in the experimental group was significantly higher than that in the control Group at one, three and six months after treatment, with a statistically significant difference (p<0.05). Table-V

**Table-V:** Comparative analysis of the negative conversion rate of sputum mycobacterium tuberculosis after treatment between the two groups (*χ̅*±*S*) n=40

Group	1 month*	3 months*	6 months*
Experimental group	19	34	38
Control group	10	25	31
c^2^	4.38	5.23	5.16
p	0.04	0.02	0.03

p<0.05

## DISCUSSION

Tuberculosis (TB), caused by Mycobacterium tuberculosis, is one of the oldest diseases known to affect humans and a leading cause of death worldwide. It is the leading cause of death in humans after HIV/AIDS.^12^ TB causes more deaths than any other infectious disease worldwide, with tuberculosis being the most frequent form.^13^ Delayed diagnosis remains a major challenge for tuberculosis control and prevention.^14^ Delayed initiation of treatment in patients with TB leads to increased infectivity, poor treatment outcomes, and increased mortality.^15^ Patients with diabetes are in a hyperglycemic state, which provides conditions for the survival of mycobacterium tuberculosis and promotes a higher incidence of tuberculosis.^16^ Patients with diabetes have a reduced ability to resist the invasion of external bacteria, aggravated islet B cell burden, and poor cellular immune function, resulting in an unsatisfactory prognosis.

Given their clinical characteristics, patients with pulmonary tuberculosis complicated with diabetes have a lengthy course of treatment and suboptimal clinical efficacy than those with pulmonary TB alone. Currently, 2HRZE/10HRE is a recommended chemotherapy regimen^17^ promoted as stabilizing the condition of patients with retreatment smear-positive pulmonary tuberculosis. It has a certain therapeutic effect but is still not ideal.^18^ Each first-line antituberculosis drug reacts adversely in a separate way. Specifically, isoniazid interferes with carbohydrate metabolism and aggravates peripheral neuritis in patients with diabetes, while rifampicin reduces the hypoglycemic effects of sulfonylureas. Ethambutol and diabetes have double adverse reactions to the eyes, which can aggravate damage to the optic nerve. Hyperglycemia can affect the blood concentration of pyrazinamide.^19^ Given the drug resistance of TB bacteria and patient dependence, there is an urgent need for a new treatment strategy to simplify and shorten the course of treatment and improve the sensitivity of anti-tuberculosis drugs.^20^

It is strongly recommended in the 2018 WHO Treatment Guidelines for Multidrug- and rifampicin-Resistant Tuberculosis (MDR/RR-TB) that levofloxacin (or moxifloxacin)^21^ be used in combination to increase treatment efficacy and reduce the development of drug resistance. Ahmad et al.^22^ believed that the combination of a new generation of fluoroquinolones had better results in the treatment of tuberculosis. Grace et al.^23^ concluded that the use of levofloxacin-containing combination chemotherapy increased treatment efficacy with no difference in adverse events compared with standard chemotherapy. Jhun et al.^24^ suggested a 6-month regimen of isoniazid, rifampicin, ethambutol, pyrazinamide, and levofloxacin (LFX), which could reduce drug resistance and increase treatment success. Lan et al.^25^ considered that a short-course treatment regimen combined with fluoroquinolones could reduce the adverse reactions of first-line anti-tuberculosis drugs.

According to the study of Serebryakova et al.^26^ It was believed that the fluoroquinolone levofloxacin had an effect on peripheral blood lymphocytes in patients with invasive pulmonary tuberculosis, and levofloxacin could increase the number of CD3 lymphocytes in patients with tuberculosis. Serebryakova et al.^27^ suggested that levofloxacin (fluoroquinolone) could inhibit the production of TNF-a in drug-resistant tuberculosis and the production of IL-12 and IFNγ in drug-sensitive tuberculosis, thereby enhancing the anti-inflammatory effect. Shah et al.^28^ confirmed that the drug concentration of levofloxacin in lung tissue was 1.71 times that of other tissues and continued to exert its efficacy for up to 120 hours. In other words, a smaller dose of levofloxacin could exert a larger therapeutic effect.^29^

It was finally confirmed in this study that the short-course chemotherapy regimen combined with levofloxacin had an efficacy of 90%, which was significantly higher than that of the control group (67.5%), with a statistically significant difference (p=0.01). After treatment, CD3^+^, CD4^+^, CD4^+^/CD8^+^ and other indicators in the experimental group were significantly higher than those in the control group, with a statistically significant difference (CD3^+^, p=0.01; CD4^+^, p=0.01; CD4^+^/CD8^+^, p=0.00); The incidence of adverse reactions was 52.5% in the experimental group and 47.5% in the control group, with no statistically significant difference (p=0.66); The negative conversion rate of patients in the experimental group was significantly higher than that in the control group at one month, three months and six months after treatment, with a statistically significant difference (p<0.05).

### Limitations of the study:

Nevertheless, shortcomings can still be seen in this study: fewer cases and short follow-up time. Moreover, no other treatment options are included for comparative analysis with this study. In response to this, more cases will be included and follow-up will continue to be extended, and other treatment regimens will be included in the study, in order to further elaborate the benefits of chemotherapy combined with levofloxacin on patients with pulmonary tuberculosis complicated with Type-2 diabetes.

## CONCLUSIONS

Chemotherapy combined with levofloxacin is a safe and effective regimen for patients with pulmonary tuberculosis complicated with Type-2 diabetes, boasting a variety of benefits such as improved clinical efficacy, ameliorated cellular immune status, a high negative conversion rate of sputum tuberculosis bacteria, and no significant increase in adverse reactions.

### Author’s Contributions:

**PL and LW:** Designed this study, prepared this manuscript, are responsible and accountable for the accuracy and integrity of the work.

**FL and YT:** Collected and analyzed clinical data, and made important contributions to the design and thinking of the study.

**LC and YL:** Data analysis, **s**ignificantly revised this manuscript.
